# Pandemic preparedness planning in peacetime: what is missing?

**DOI:** 10.1186/s42522-020-00027-2

**Published:** 2020-08-14

**Authors:** Ab Osterhaus, John Mackenzie

**Affiliations:** 1 Research Center Emerging Infections and Zoonoses (RIZ) University of Veterinary Medicine Hannover, Hanover, Germany; 2 Faculty of Health Sciences, Curtin University, Perth, Western Australia; 3 School of Chemistry and Molecular Biosciences, The University of Queensland, Brisbane, Queensland Australia

There have been two major novel disease outbreaks of international concern in the past two decades, ‘swine flu’ or ‘Mexican flu’ in 2009–10 and SARS-coronavirus (SARS-CoV) in 2002–03. SARS-CoV spread to 29 countries in three Continents, whereas the ‘swine flu’ spread globally as a pandemic, affecting all countries around the World. These two outbreaks clearly demonstrated that:

- A previously unknown pathogen could emerge from an unknown source at any time and in any place and, without warning, threatening the health, well-being and economies of all societies;
- There is a clear need for countries to have the capability and capacity to maintain an effective alert and response system to detect and quickly react to outbreaks of international concern, and to share information about such outbreaks rapidly and transparently
- Responding to pandemic threats requires global cooperation and global participation [1].

Although the last influenza pandemic of 2009–10, should probably be ranked as moderate in terms of morbidity and mortality on the scale of pandemics [2], it could have been expected that it, together with the SARS outbreak [3], should have functioned as a wake-up call for better pandemic preparedness, regardless of virus species or zoonotic source. In addition, outbreaks of animal disease with pandemic potential, such as highly pathogenic avian influenza H5N1viruses first discovered in fatal human cases in 1997 [4], caused many outbreaks in poultry and high fatality rates in humans, with the human cases reported from 17 countries in Asia, Africa, the Pacific, Europe and the Near East since November 2003 [5]. Thus, in the interpandemic decade since 2010, the need for pandemic preparedness was generally not appreciated to the full extent, despite strong country support for influenza pandemic planning provided by the World Health Organization (WHO). Thus most planning was predicated on a pandemic being caused by an influenza virus, and not by viruses belonging to a different genus or family. Nevertheless, it may be concluded that measures to provide better global health security in terms of pandemic preparedness, should have deserved more attention. Now, whilst in the middle of COVID-19 pandemic crisis, there is a growing awareness of shortcomings of pandemic preparedness at national and international levels.

As all pandemic viruses that emerged during the last century had their origin in the animal world, and ongoing changes in the respective interfaces between humans, animals and the environment have led to an increase in major predisposing factors that allow the emergence of zoonotic viruses as novel human pathogens, pandemic preparedness measures in “peacetime” should follow a “One Health” approach [6].

Therefore, the most logical measures would focus on limiting the upward trend of pandemic threats by reducing the increase of these major predisposing factors. However, as most of these are probably hard to rollback in our modern society [7], we should in addition profit more from our most recent scientific, technical and clinical achievements.

Among the pandemic preparedness measures, at least the following elements should be represented:

- **Early warning systems** that are based on syndrome- and laboratory-based surveillance and associated reporting for humans and animals alike, should be in place. This should involve medical and veterinary professionals working in close collaboration with experts in laboratory-based diagnostics and rapid molecular virus characterization, with close links to national and international reporting systems [8]. Additional information may be obtained from filtered national and local news gatherings that signal elevations in human and animal infectious disease morbidity or mortality patterns. Based on these principles national and international surveillance systems for respiratory, enteric and neurological infections in humans have been established in numerous countries. However, international and global coverage, collaboration, coordination and willingness to share data and report newly emerging threats in a timely fashion, according to e.g. the WHO international health regulations (IHR) requirements [9], are not always optimal. Similarly, syndrome and laboratory surveillance for domestic and wild animals with close links to national and international reporting systems coordinated by the Organization for Animal Health (OIE) and Food and Agriculture Organization (FAO) [10], should form the basis for animal health security, and preparedness for the unprecedented epidemic or pandemic spread of animal diseases like avian influenza and African swine fever. This preparedness should also form the basis of the early warning for zoonotic threats with pandemic potential. It should however be noted that viruses with zoonotic, epidemic or even pandemic potential for humans do not necessarily cause major signs or symptoms in animals. Again, global coverage, international collaboration, coordination and willingness to share data and report newly emerging threats in a timely fashion, according to OIE and FAO requirements are not always optimal. Indeed, few countries undertake broadly-based, real-time animal disease surveillance; most animal disease detection is only recognized by obvious animal die-offs, or by research interests in specific animal diseases. Good and sustained animal diseases surveillance is urgently needed, and needs to be integrated with human disease surveillance. This is particularly important in many resource-poor settings, which would require assistance from international philanthropic organizations.
- Early identification of an emerging pathogen with pandemic potential, is of utmost importance to limit its initial spread, buying the world time to prepare while crucial information on the nature of the novel pathogen is disseminated. This implies that state-of-the-art **pathogen discovery and characterization platforms** [11], which are operational predominantly in networks of specialized laboratories with at least BSL3 facilities, should be involved from the very beginning in outbreak management scenarios. In recent years molecular techniques like next generation sequencing (NGS), combined with the associated bioinformatics platforms have allowed the identification and initial characterization of novel pathogens and their receptors, within a matter of weeks or even days [12, 13]. This was noted in the current COVID-19 pandemic [14] and raised the question of the added value of Koch’s postulates in the current era of NGS. In comparison, the discovery of e.g. HIV and its primary receptor in the eighties of the last century, rather took years after the initial identification of AIDS as a new disease entity [15]. A global collaborative infrastructure, based on trust and compliance, that allows early exchange of information and materials, while protecting the interest of all parties involved from the beginning, is of crucial importance to achieve this goal.
- With the rapidly acquired knowledge about the nature and molecular characteristics of a newly identified pandemic virus and once the relevant clinical samples are accessible, **diagnostic methods** can be developed in a matter of days, using existing diagnostic platforms [16]. These methods can be disseminated and used for individual patient diagnosis and isolation or quarantine measures, as well as for epidemiological studies to monitor the spreading of the pandemic virus. Furthermore, the rapid identification of individuals with the infection allows the gathering of data on clinical manifestations, pathogenesis and pathology of the infection as it spreads. This will not only be of help for the early identification and isolation of infected individuals, but also for the development of effective therapeutic and preventive intervention strategies.
- The rapid acquisition of data on clinical manifestations, pathogenesis, molecular viral phylogeny, transmission, epidemiology, treatment and other intervention options will also feed early **mathematical models** that may help to determine the expected course of the pandemic geographically and in time [17]. These models also can be instrumental in helping to design, compare and select pharmaceutical as well as non-pharmaceutical intervention strategies. Specialized expertise in establishing mathematical models for these purposes should be considered important from the very start of a pandemic.
- To prove that a newly identified virus is indeed the cause of an emerging pandemic by establishing the Koch postulates and also as an aid to study its associated pathology, pathogenesis and ways of transmission, the ability to rapidly establish **animal models in BSL3 facilities** is of crucial importance [18], but as noted above, identification of a novel pathogen by new technologies such as NGS should be applied prior to isolation of a novel organism. Such animal models can be selected on basis of proximity to humans, suspected animal origin of the virus, and preliminary information on the pandemic virus like e.g. its receptor usage. Once animal models are established, they also can be used for preclinical testing of pharmaceutical and non-pharmaceutical intervention strategies.
- Besides preclinical in vitro and in vivo test facilities to evaluate intervention strategies, the availability of experienced **clinical trial organisations** to conduct Phase I, II and III trials in the shortest period of time in close consultation with regulatory authorities, is essential to evaluate non-pharmaceutical as well as pharmaceutical intervention strategies, like candidate antivirals and vaccines, as they become available.
- To reduce the spread of the virus both **non-pharmaceutical and pharmaceutical intervention strategies** need to be developed and implemented as soon as possible according to preparations made in “peacetime”:
  - Implementation of **non-pharmaceutical intervention** strategies largely depends on preparations that have been made in “peacetime” as well as the nature of the virus and its potential and ways to spread. This includes stockpiling of devices such as mouth masks, protective clothing and other protective materials for the public at large.
  - Platforms should be developed for the rapid development of **broadly protective anti-viral vaccines** that can either be produced directly or after further adaptation to the newly emerged pandemic virus [19]. Such vaccines should preferably provide protection against several members of the same virus family, like e.g. more universal vaccines against influenza- or corona viruses. It should be realized however, that there are several other virus families that harbor viruses with pandemic potential. To achieve this goal, studies towards understanding correlates of broad protection within these families should be undertaken, together with studies exploring vaccine platforms that are designed to induce such protective responses. The discovery and development of such vaccines have been stimulated by different funding initiatives in recent years, but given their importance for better pandemic preparedness, a more solid investment in this research area should be warranted.
  - **Existing vaccines** against non-related pathogens, like BCG, Polio- or MMR vaccine, have been used to boost innate immunity as a measure to mitigate the outcome of a pandemic virus infection. Their potential value needs further confirmation in clinical trials [20].
- To treat infected persons, which may also contribute to reduction of virus spread, several strategies should be followed from the beginning of a pandemic. Adequate infection protection for healthcare workers should be ensured during patient treatment at all times.
  - Sufficient supplies of personal protection equipment (PPE) for healthcare workers is essential, and stockpiles of PPE should be procured during ‘peacetime’, with agreements in place for the supplies of PPE during a pandemic
  - At the beginning of a pandemic, therapeutic interventions have to rely on **symptomatic treatment** in first-line practice, regular hospitals and ICUs. Treatment largely depends on accumulated data on the observed symptomatology, pathogenesis and pathology as the pandemic progresses. Pre-pandemic preparedness will have to secure sufficient personnel and equipment capacities at all these levels - if possible - without infringing on regular hospital routine for non-pandemic patients.
  - Sufficient supplies and production capacity for **antibiotics** against secondary bacterial infections should be secured.
  - Platforms for **repurposing of existing antivirals** developed against other viruses, based on shared working mechanisms, should be used to test their suitability for treatment of pandemic cases, by using in vitro and in vivo evaluation systems before clinical trials are started.
  - Platforms should be used for the rapid development of **broadly protective anti-viral drugs** that can either be produced directly or after an adaptation to the newly emerged pandemic virus [21]. Such antivirals could provide protection against several members of the same virus family, like e.g. more universal antivirals against influenza or corona viruses. As for vaccines, it should be realized however, that there are several virus families that harbor viruses with pandemic potential.
  - Platforms should be used for the rapid development of **broadly protective human monoclonal antibodies** that can either be produced directly or after an adaptation to the newly emerged pandemic virus [22]. Such antiviral antibodies could provide protection against several members of the same virus family, like e.g. more universal human antibodies against influenza or corona viruses. In the absence of broadly protective human monoclonal antibodies, the use of **human convalescent plasma antibodies** may be considered [23].
  - Platforms should be used to test the efficacy of **biological response modifiers** as an adjunct to the treatment of clinical cases. This should be based on accumulating data about the natural history and insights in the pathogenesis of the infection and could include the use of e.g. interferon inducers and corticosteroids [24].
- Strategies to ensure adequate mutual communication between scientists, policymakers and the public is of utmost importance to ensure support for the implementation of all policy measures intended to efficiently combat the pandemic as it spreads.

On the basis of at least a combination of the “peacetime” preparations mentioned above, the development of internationally coordinated pandemic preparedness plans involving closely collaborating national and international health organizations like WHO, OIE and FAO should be a priority to limit the burden of, or eventually even prevent future pandemics. This should lead to the adequate international coordination from early warning with options of early control, to arrangements for the sharing of information and scientific knowledge, eventually leading to the equitable distribution of live saving vaccines and drugs.
